# Cell Wall Profiling of the Resurrection Plants *Craterostigma plantagineum* and *Lindernia brevidens* and Their Desiccation-Sensitive Relative, *Lindernia subracemosa*

**DOI:** 10.3390/plants13162235

**Published:** 2024-08-12

**Authors:** John P. Moore, Brock Kuhlman, Jeanett Hansen, Leonardo Gomez, Bodil JØrgensen, Dorothea Bartels

**Affiliations:** 1 South African Grape and Wine Research Institute, Department of Viticulture and Oenology, Faculty of AgriSciences, Stellenbosch University, Stellenbosch 7600, South Africa; brock.kuhlman@gmail.com; 2 Department of Plant and Environmental Sciences, University of Copenhagen, 1871 Copenhagen, Denmark; jeanetthansen73@gmail.com (J.H.); boj@plen.ku.dk (B.J.); 3 Centre of Novel Agricultural Products (CNAP), Department of Biology, University of York, Heslington, York YO10 5DD, UK; leonardo.gomez@york.ac.uk; 4 IMBIO, University of Bonn, Kirschallee 1, D-53115 Bonn, Germany; dbartel1@uni-bonn.de

**Keywords:** *Craterostigma*, *Lindernia*, evolution, glycan microarrays, monosaccharides, cell walls

## Abstract

Vegetative desiccation tolerance has evolved within the genera *Craterostigma* and *Lindernia*. A centre of endemism and diversification for these plants appears to occur in ancient tropical montane rainforests of east Africa in Kenya and Tanzania. *Lindernia subracemosa*, a desiccation-sensitive relative of *Craterostigma plantagineum*, occurs in these rainforests and experiences adequate rainfall and thus does not require desiccation tolerance. However, sharing this inselberg habitat, another species, *Lindernia brevidens*, does retain vegetative desiccation tolerance and is also related to the resurrection plant *C. plantagineum* found in South Africa. Leaf material was collected from all three species at different stages of hydration: fully hydrated (ca. 90% relative water content), half-dry (ca. 45% relative water content) and fully desiccated (ca. 5% relative water content). Cell wall monosaccharide datasets were collected from all three species. Comprehensive microarray polymer profiling (CoMPP) was performed using ca. 27 plant cell-wall-specific antibodies and carbohydrate-binding module probes. Some differences in pectin, xyloglucan and extension epitopes were observed between the selected species. Overall, cell wall compositions were similar, suggesting that wall modifications in response to vegetative desiccation involve subtle cell wall remodelling that is not reflected by the compositional analysis and that the plants and their walls are constitutively protected against desiccation.

## 1. Introduction

Resurrection plants are highly adapted to withstand desiccation tolerance and can revive from vegetative tissue with a relative water content of less than 10% or a water potential of less than −100 MPa [1]. Present on all continents except Antarctica, resurrection plants have been able to pioneer extreme environments such as inselbergs, which experience periodic or seasonal drying [2,3]. On the African continent, these plants are present in deserts and semi-deserts to montane rainforests and can have a broad geographic range or be highly localised [3,4].

*Craterostigma plantagineum*, a dicotyledonous plant, covers a large area from East Africa extending into Niger, Sudan, Ethiopia and Southern Africa that is characterised by restricted water availability and a low-to-moderate elevation. In contrast, *Lindernia brevidens*, a related desiccation-tolerant plant described in 2008, is localised to the montane rainforests of Tanzania and Kenya, which experience high precipitation (>1500 mm per year). A close relative of *L. brevidens*, *L. subracemosa*, which occupies a similar habitat in western Tanzania, Kenya, Eastern Congo and Ethiopia, is desiccation-sensitive [4]. All three species belong to the family Linderniaceae and are defined taxonomically as being closely related in Phillips et al. (2008) [4].

Resurrection plants use various strategies to achieve desiccation tolerance, which can be constitutive or inducible and involve changes to primary and secondary metabolites [3]. The plant cell wall, comprised mainly of cellulose, hemicellulose and pectin polymers, constitutes a significant proportion of the cell biomass and is responsible for maintaining structural integrity. Therefore, any strategies for desiccation tolerance will likely incorporate dynamic changes to the cell wall to preserve cell integrity during periods of desiccation and subsequent hydration [5]. In *Oryza sativa*, cell wall irreversibility through the build-up of secondary cell wall components has been identified as a limiting factor in drought tolerance, highlighting the dynamic nature of the changes that occur in the cell wall during water-deficit stress [6]. Understanding these mechanisms in resurrection plants will expand the repertoire available to researchers in the development of drought-tolerant crop plants and novel cell-wall-based biomaterials [7].

The desiccation of plant vegetative tissues leads to cell plasmolysis, which generates mechanical stress. In resurrection plants, such mechanical stress can be reduced through vacuolation and reversible cell wall folding. The latter involves dynamic changes of the cell wall polysaccharide composition. These changes include the increased presence of de-methylesterified homogalacturonan upon desiccation, which has been proposed to strengthen the cell wall. In *C. plantagineum*, xyloglucan levels have been shown to increase upon desiccation, which is thought to provide increased strength. Furthermore, changes to rhamnogalacturonan-II levels have also been reported for *C. plantagineum* [8]. These changes are required to enable reversible cell wall expansion and folding during desiccation. Cell wall folding has also been reported in *L. brevidens* and *L. subracemosa*, in spite of the latter being desiccation-sensitive [9]. The literature provides limited information on which of the changes that occur in the cell wall composition and architecture during desiccation and hydration are associated with desiccation tolerance. In the present study we compare the cell wall profiles of two desiccation-tolerant plants, *C. plantagineum* and *L. brevidens*, with the desiccation-sensitive *L. subracemosa*, with the aim of establishing which wall features determine desiccation tolerance.

## 2. Results

### 2.1. Analysis of Cell Wall Composition

**Figure 1:**
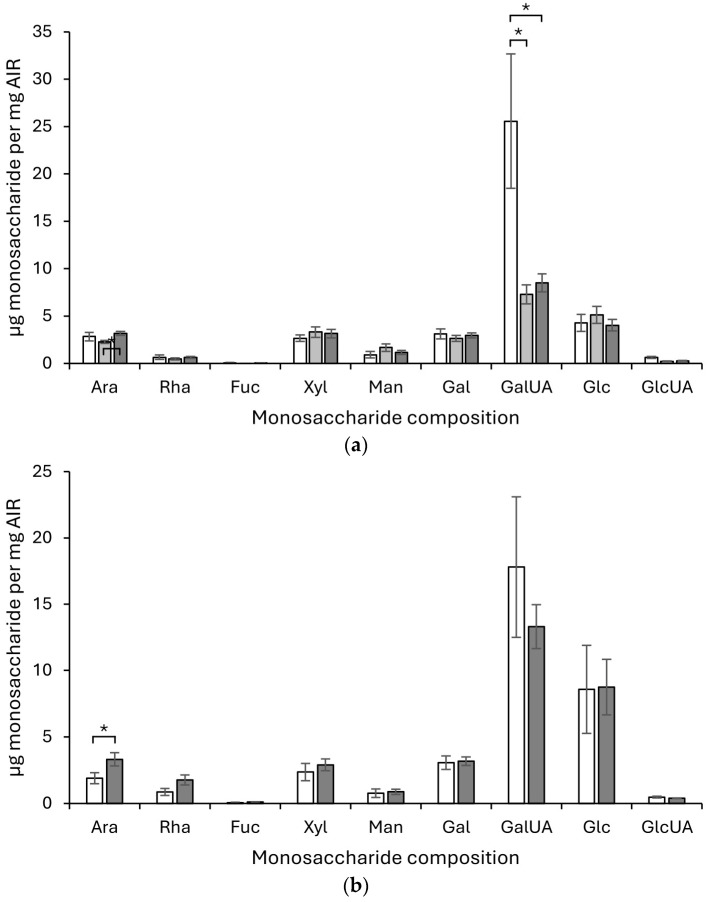
Monosaccharide compositional analysis of the total AIR isolated from leaf material of *Craterostigma plantagineum* (**a**), and *Lindernia brevidens* (**b**). White bars represent hydrated leaves, mid-grey shaded bars represent partially hydrated leaves and shaded bars represent desiccated leaves. Monosaccharide codes are for arabinose (Ara), rhamnose (Rha), fucose (Fuc), xylose (Xyl), mannose (Man), galactose (Gal), galacturonic acid (GalUA), glucose (Glc) and glucuronic acid (GlcUA). Error bars represent the standard error (SE) of the mean of four biological samples with two technical replicates per biological sample. Statistically significant differences, based on one-way ANOVA variance testing, are indicated on the bar graphs as an asterisk.

The total monosaccharide composition of cell wall fractions was analysed for *C. plantagineum* and *L. brevidens* using the alcohol-insoluble residue (AIR) isolated from leaf material that was hydrated, with a relative water content (RWC) ~ca. 90%; partially hydrated, with an RWC~ ca. 45% (in the case of *C. plantagineum*); and desiccated, with an RWC ~ca. 5% (Figure 1). For *C. plantagineum*, the main monosaccharides detected were GalUA, Glc, Xyl, Gal and Ara, indicating that the cell wall is pectin-rich (Figure 1a). Significant changes in the main monosaccharides between hydration states were detected for Ara between the partially hydrated and desiccated states and for GalUA between the hydrated state and the partially hydrated and desiccated states. Ara concentrations increased from the partially hydrated to the desiccated state. GalUA concentrations decreased considerably from the hydrated state to the partially hydrated and desiccated states. No significant differences in Glc, Xyl and Gal were observed.

For *L. brevidens*, the main monosaccharides detected were also GalUA, Glc, Gal, Xyl and Ara, indicating that the cell wall is also pectin-rich (Figure 1b). Significant changes in the main monosaccharides between hydration states were only detected for Ara, which increased its concentration from the hydrated state to the dehydrated state. No significant differences in GalUA, Glc, Gal and Xyl were detected. The ratios of the different monosaccharides present in the cell wall for *C. plantagineum* and *L. brevidens* were the same. Insufficient AIR material was available from *L. subracemosa* to obtain monosaccharide datasets. Starch-derived glucose in the AIR cannot be excluded from consideration in the results obtained. Saccharification analysis of the AIR sourced from the different species (see Supplementary Datasets) was performed; however, no clear patterns were discernible.

### 2.2. CoMPP Analysis of Cell Wall Material

**Figure 2:**
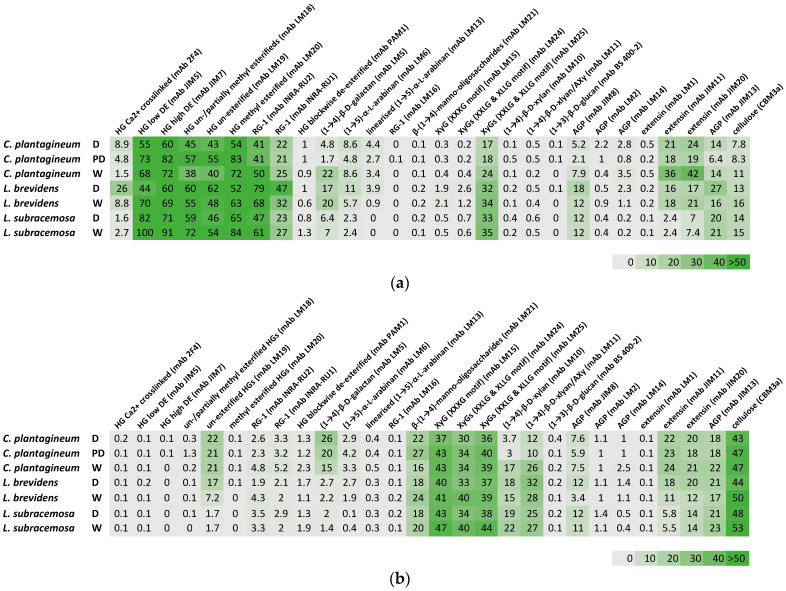
Comprehensive microarray polymer profiling (CoMPP) analysis of plant-leaf cell wall fractions from CDTA-extractable material (**a**) and NaOH-extractable material (**b**) isolated from *Craterostigma plantagineum*, *Lindernia brevidens* and *Lindernia subracemosa* leaves that were hydrated (H), partially hydrated (PD) or desiccated (D). The heatmaps indicate the relative abundance of plant cell wall glycan-associated epitopes present in the AIR, and the colour intensity is correlated to the mean spot signals. The values in the heatmap are the mean spot signals from three experiments. The highest signal in the entire data set was set to 100, and all other data were adjusted accordingly.

Plant-leaf cell wall fractions from CDTA-extractable material and NaOH-extractable material isolated from the three species at different hydration states were compared (Figure 2). All three species, *C. plantagineum* and *L. brevidens* and the desiccation-sensitive *L. subracemosa*, demonstrated a high abundance of homogalacturonan (HG), rhamnogalacturonan (RG-I) and cellulose. Moderate levels of xyloglucan (XyG) epitopes and epitopes for manno-oligosaccharides, xylan, extensin and arabinogalactan protein (AGP) were detected. Arabinan epitopes were detected at low levels, while the glucan epitope (mAb BS 400-2) was not present. A galactan epitope was present at moderate levels in *C. plantagineum* and *L. brevidens* but not in *L. subracemosa*.

In *C. plantagineum*, inspection of the datasets suggest that the levels of HG epitopes varied upon desiccation. Calcium ion-crosslinked HG levels (mAb 2F4) increased, and levels of partially unesterified and esterified HG (mAbs LM18 and LM19) were slightly elevated. In contrast, levels of high- and low-DE HG (mAbs JIM5 and JIM7) and esterified HG (mAb LM20) were decreased upon desiccation. Blockwise, de-esterified HG (mAb PAM1) was not detected. Levels of detectable RG-I epitopes (mAbs INRA-RU1 and INRA-RU2) decreased slightly in response to desiccation. Levels of galactan (mAb LM5) were found to increase in the CDTA-extracted material and decrease in the NaOH-extracted material upon desiccation. Levels of arabinan (mAbs LM6 and LM13) remained fairly constant irrespective of their hydration status. Levels of manno-oligosaccharides (mAb LM 21) were also slightly elevated in desiccated *C. plantagineum*. A small decrease was observed for xyloglucan (mAbs LM15, LM24 and LM25) and AGP (mAbs JIM8, LM2, LM14 and JIM13) between hydration states. In contrast, there was a decrease in the levels of xylan (mAb LM10) and arabinoxylan (AX, mAb LM11) upon desiccation. A decrease in the levels of extensin (mAbs JIM11 and JIM 20) was detected in the CDTA-extracted material but not in the NaOH-extracted material. The levels of cellulose or xyloglucan (mAbs CBM3a) decreased slightly in response to desiccation.

Similar changes in HG epitope levels were observed for *L. brevidens*. In contrast, levels of detectable RG-1 epitopes (mAbs INRA-RU1 and INRA-RU2) were seen to increase in response to desiccation. No major change in galactan (mAb LM5) levels were observed. In contrast to *C. plantagineum*, in *L. brevidens*, the levels of arabinan (mAbs LM6 and LM13) were seen to increase slightly in response to desiccation, while manno-oligosaccharide (mAb LM 21) levels declined slightly. Similar to *C. plantagineum*, a small decrease was observed for xyloglucan (mAbs LM15, LM24 and LM25). Xylan (mAb LM10), arabinoxylan (AXy, mAb LM11) and AGP (mAbs JIM8, LM2, LM14 and JIM13) increased slightly upon desiccation in contrast with the change in levels seen in *C. plantagineum*. A decrease in the levels of extensin (mABs JIM11 and JIM 20) was detected. The levels of cellulose (mAbs CBM3a) also decreased slightly in response to desiccation.

For the desiccation-sensitive *L. subracemosa*, changes in HG epitope levels were similar to those in the resurrection plants, except for the levels of partially unesterified and esterified HG (mAbs LM18 and LM19), which were found to decrease in response to desiccation. Levels of detectable RG-1 epitopes (mAbs INRA-RU1 and INRA-RU2) were seen to increase, similarly to that observed for *C. plantagineum*. In contrast to the resurrection plants, *L. subracemosa* had lower levels of galactan (mAb LM5) and arabinan (mAbs LM6 and LM13) overall, and these levels did not change in response to desiccation. Levels of manno-oligosaccharides (mAb LM 21) declined slightly with desiccation. Similarly to the resurrection plants, there was a small decrease in the levels of xyloglucan (mAbs LM15, LM24 and LM25). Xylan (mAb LM10) and arabinoxylan (AXy, mAb LM11) levels decreased slightly upon desiccation, similarly to *C. plantagineum*. Levels of extensin (mABs JIM11 and JIM 20) and AGP (mAbs JIM8, LM2, LM14 and JIM13) remained constant, in contrast to the changes observed in the resurrection plants.

## 3. Discussion and Conclusions

The first detailed cell wall analysis of an angiosperm resurrection plant was reported by Vicré and colleagues on *Craterostigma wilmsii *[10,11]. The initial study was a microscopic and immunocytochemical analysis [10] followed by a biochemical analysis of the cell walls at different states of hydration or desiccation [11] These studies implicated unesterified pectins and xyloglucan as well as substitutional changes in the hemicellulose fraction as factors important upon desiccation [10,11]. A later study on *Craterostigma plantagineum* plants suggested that expansins may be upregulated upon desiccation and play a role in the vegetative desiccation tolerance of resurrection plants [12]. Moore et al. proposed RG-I linked arabinans and arabinogalactan proteins as “pectic plasticizers” that protect the cell wall of the southern African resurrection plant species *Myrothamnus flabellifolia* against mechanical stresses induced by complete dehydration [13]. Moore et al. surveyed a range of southern African resurrection plant species using CoMPP technology [14]. The study proposed that potential cell wall strengthening mechanisms had evolved in parallel with angiosperm evolution, i.e., that vegetative desiccation tolerance re-evolved as a genetic trait in different lineages over evolutionary time [14,15,16]. In relation to our results, a comparison can be made with respect to the Moore et al. (2013) study [14], where GalUA levels were elevated in both hydrated and desiccated plants, versus this report, where they were found to be only elevated in hydrated leaves of *C. plantagineum*. The significance of why this is the case is unclear. Galactan differences were also found, but the importance of this is uncertain. Other noteworthy differences are the presence of more extension epitopes in both desiccation-tolerant species, which seemed to increase upon hydration in *C. plantagineum*. Again, the significance of this is unclear. Pectin esterification levels also changed in *C. plantagineum* in a similar manner to that observed by Jung et al. [9], whose study focused on a glycine-rich protein forming complexes with pectin during desiccation and recovery. A recent review of vegetative desiccation tolerance and resurrection plant cell wall changes proposed a glycine-rich protein–wall-associated kinase–arabinogalactan protein–FERONIA–pectin complex as crucial in releasing stored calcium ions during desiccation for release upon rehydration, facilitating ”resurrection” in these remarkable species [9,16,17]. This study revealed that in all three species, no major changes in cell wall composition were detected using the profiling technologies employed. This is not to say that there are no significant changes occurring as subtle changes are more likely, which suggests that wholesale reorganisation of the cell wall in these plants is not a requirement for surviving desiccation. This would indicate that more efforts should be employed in the evaluation of wall–membrane signalling processes during dehydration to elucidate the mechanisms by which these factors contribute to vegetative desiccation tolerance in angiosperm resurrection plants.

## 4. Materials and Methods

### 4.1. Plant Material

*Lindernia brevidens* and *Lindernia subracemosa* were originally collected from the Taita Hills, Kenya, and then propagated at the IMBIO lab of the University of Bonn (Germany). Four voucher specimens were deposited at Koblenz University (Germany). *L. brevidens* seeds were germinated directly in potting compost and maintained in a climate chamber at day/night temperatures of 22 °C and 18 °C. Plants were grown under a 16 h day/8 h night regime in the growth chambers at the IMBIO, University of Bonn (Germany). *Craterostigma plantagineum* plants were collected and grown as described previously [18]. Plants were gradually dried in pots over a period of 12–21 days. Relative water content measurements were determined according to the method described previously [14]. Four biological samples with two technical replicates per biological sample were conducted for all analyses in this brief study.

### 4.2. Isolation of Cell Wall Material

Lyophilised leaf material was ground to a fine powder under liquid nitrogen using a Retsch Mixer-Mill (Retsch, Haan, Germany). Powdered lyophilates were suspended in boiling 80% (*v*/*v*) aqueous ethanol for 15 min to deactivate any endogenous enzymes present. A series of organic solvent extractions were performed to remove low-molecular-weight metabolites from the cell-wall-containing residues. Residues were extracted for 2 h at room temperature: twice with methanol–chloroform (1:1, *v*/*v*), twice with methanol–acetone (1:1, *v*/*v*), and finishing with acetone–water (4:1, *v*/*v*). The residues were then freeze-dried (Christ Lyophilizer, Martin Christ Gefriertrocknungsanlagen GmbH, Osterode am Harz, Germany). Solvent reagents were obtained from Sigma-Aldrich, Johannesburg, South Africa.

### 4.3. Saccharification Analysis

Saccharification efficiency was determined following the method described by Gómez and coauthors at the Centre for Novel Agricultural Products (CNAP) [19]. Ground material was weighed into 96-well plates using a custom-made robotic platform (Labman Automation, Stokesley, North Yorkshire, UK), with each well containing 4 mg of each sample. Pretreatment, enzymatic hydrolysis and sugar determination were performed automatically by a robotic platform (Tecan Evo 200; Tecan Group Ltd., Männedorf, Switzerland). The amount of released sugars was assessed against a glucose standard curve using the 3-methyl-2-benzothiazolinone hydrozone method (MTBH, Sigma-Aldrich, Gillingham, UK) [20].

### 4.4. Monosaccharide Composition of Cell Walls

Cell wall monosaccharide analysis was performed as described previously [21]. Cell walls were prepared by homogenising plant materials in liquid phenol and washing with chloroform:methanol (2:1) before sedimentation by centrifugation. The pellets were washed twice with 95% ethanol and left to dry. To analyse the monosaccharide content of non-cellulosic polysaccharides, wall material was hydrolysed with 2 M TFA for 4 h at 100 °C before separation by high-performance anion-exchange chromatography (HPAEC) on a CarboPac PA-20 column with pulsed amperometric detection. Separated monosaccharides were quantified by external calibration using an equimolar mixture of monosaccharide standards, which had also been treated with 2 M TFA in the same way.

### 4.5. CoMPP Analysis of Cell Wall Material

CoMPP (comprehensive microarray polymer profiling) involves the use of monoclonal antibodies and carbohydrate-binding modules to detect plant cell-wall-specific epitopes as detailed previously [22]. The AIR was extracted sequentially using an aqueous 50 mM 1,2-cyclo-hexylene-dinitrilo-tetra-acetic acid (CDTA; Merck Darmstadt, Germany) solution followed by a 4 M aqueous NaOH solution (Merck Darmstadt, Germany). Each fraction was spotted onto a nitrocellulose membrane using an Arrayjet printer (Marathon, Arrayjet, Edinburgh, UK). Individual microarrays were probed with a list of specific antibodies and other probes (see Supplementary Table S1 for probes used and references) and developed using buffered aqueous 5-bromo-4-chloro-3-indolyl phosphate and nitrotetrazolium blue solution (Merck, Darmstadt, Germany). Microarrays, after drying, were scanned using a flatbed scanner (Canon 8800, Søborg, Denmark) and then processed using array detection software (Array-Pro Analyzer v 6.3, MediaCybernetics, Rockville, MD, USA). The result was displayed as a heatmap using Excel software (Version 2407) (Microsoft, Redmond, Washington, DC, USA).

### 4.6. Statistical and Univariate Tools

Statistical analyses were performed in consultation and collaboration with Professor Martin Kidd of the Centre for Statistical Consultation (Stellenbosch University). Descriptive statistical analyses and analysis of variance (ANOVA) were performed with the statistical packages of Microsoft Excel (Microsoft, Redmond, Washington, DC, USA) and Statistica (version 14.0.1.25) (Statsoft, Southern African Analytics Pty Ltd., Johannesburg, South Africa) software.

## Data Availability

Data are available upon request from the corresponding author.

## Supplementary Materials

The following supporting information can be downloaded at: https://www.mdpi.com/article/10.3390/plants13162235/s1, Supplementary Table S1: Raw datasets—Moore et al. 2024 Plants monosacch. raw datasets.xlsx and Moore et al. 2024 Plants CoMPP raw datasets.xlsx. Refs. [23,24,25,26,27,28,29,30,31,32,33,34,35,36] are cited in the Supplementary Materials.

## References

[1] Marks R.A., Pas L.V.D., Schuster J., Gilman I.S., VanBuren R. (2024). Convergent Evolution of Desiccation Tolerance in Grasses. Nat. Plants.

[2] VanBuren R., Wai C.M., Giarola V., Župunski M., Pardo J., Kalinowski M., Grossmann G., Bartels D. (2023). Core Cellular and Tissue-Specific Mechanisms Enable Desiccation Tolerance in Craterostigma. Plant J..

[3] Djilianov D., Moyankova D., Mladenov P., Topouzova-Hristova T., Kostadinova A., Staneva G., Zasheva D., Berkov S., Simova-Stoilova L. (2024). Resurrection Plants—A Valuable Source of Natural Bioactive Compounds: From Word-of-Mouth to Scientifically Proven Sustainable Use. Metabolites.

[4] Phillips J.R., Fischer E., Baron M., Van Den Dries N., Facchinelli F., Kutzer M., Rahmanzadeh R., Remus D., Bartels D. (2008). *Lindernia Brevidens*: A Novel Desiccation-Tolerant Vascular Plant, Endemic to Ancient Tropical Rainforests. Plant J..

[5] Moore J.P., Vicré M., Nguema-Ona E., Driouich A., Farrant J.M. (2023). Drying Out Walls: How Do the Cell Walls of Resurrection Plants Survive Desiccation?. Plant Cell Walls.

[6] Ilias I.A., Wagiran A., Azizan K.A., Ismail I., Samad A.F.A. (2024). Irreversibility of the Cell Wall Modification Acts as a Limiting Factor in Desiccation Tolerance of *Oryza Sativa* Ssp. *Indica* Cv MR303. Plant Stress.

[7] Xu X., Legay S., Sergeant K., Zorzan S., Leclercq C.C., Charton S., Giarola V., Liu X., Challabathula D., Renaut J. (2021). Molecular Insights into Plant Desiccation Tolerance: Transcriptomics, Proteomics and Targeted Metabolite Profiling in *Craterostigma plantagineum*. Plant J..

[8] Chen P., Jung N.U., Giarola V., Bartels D. (2020). The Dynamic Responses of Cell Walls in Resurrection Plants During Dehydration and Rehydration. Front. Plant Sci..

[9] Jung N.U., Giarola V., Chen P., Knox J.P., Bartels D. (2019). *Craterostigma plantagineum* Cell Wall Composition Is Remodelled during Desiccation and the Glycine-Rich Protein CpGRP1 Interacts with Pectins through Clustered Arginines. Plant J..

[10] Vicré M., Sherwin H.W., Driouich A., Jaffer M.A., Farrant J.M. (1999). Cell Wall Characteristics and Structure of Hydrated and Dry Leaves of the Resurrection Plant *Craterostigma Wilmsii*, a Microscopical Study. J. Plant Physiol..

[11] Vicré M., Lerouxel O., Farrant J., Lerouge P., Driouich A. (2004). Composition and Desiccation-Induced Alterations of the Cell Wall in the Resurrection Plant *Craterostigma wilmsii*. Physiol. Plant.

[12] Jones L., McQueen-Mason S. (2004). A Role for Expansins in Dehydration and Rehydration of the Resurrection Plant *Craterostigma plantagineum*. FEBS Lett..

[13] Moore J.P., Nguema-Ona E., Chevalier L., Lindsey G.G., Brandt W.F., Lerouge P., Farrant J.M., Driouich A. (2006). Response of the Leaf Cell Wall to Desiccation in the Resurrection Plant *Myrothamnus flabellifolius*. Plant Physiol..

[14] Moore J.P., Nguema-Ona E.E., Vicré-Gibouin M., Sørensen I., Willats W.G.T., Driouich A., Farrant J.M. (2013). Arabinose-Rich Polymers as an Evolutionary Strategy to Plasticize Resurrection Plant Cell Walls against Desiccation. Planta.

[15] Moore J.P., Vicré-Gibouin M., Farrant J.M., Driouich A. (2008). Adaptations of Higher Plant Cell Walls to Water Loss: Drought vs Desiccation. Physiol. Plant.

[16] Dace H.J., Adetunji A.E., Moore J.P., Farrant J.M., Hilhorst H.W. (2023). A Review of the Role of Metabolites in Vegetative Desiccation Tolerance of Angiosperms. Curr. Opin. Plant Biol..

[17] Chen P., Giarola V., Bartels D. (2021). The *Craterostigma plantagineum* Protein Kinase CpWAK1 Interacts with Pectin and Integrates Different Environmental Signals in the Cell Wall. Planta.

[18] Bartels D., Schneider K., Terstappen G., Piatkowski D., Salamini F. (1990). Molecular Cloning of Abscisic Acid-Modulated Genes Which Are Induced during Desiccation of the Resurrection Plant *Craterostigma plantagineum*. Planta.

[19] Gomez L.D., Whitehead C., Barakate A., Halpin C., McQueen-Mason S.J. (2010). Automated Saccharification Assay for Determination of Digestibility in Plant Materials. Biotechnol. Biofuels.

[20] Anthon G.E., Barrett D.M. (2002). Determination of Reducing Sugars with 3-Methyl-2-Benzothiazolinonehydrazone. Anal. Biochem..

[21] Jones L., Milne J.L., Ashford D., McQueen-Mason S.J. (2003). Cell Wall Arabinan Is Essential for Guard Cell Function. Proc. Natl. Acad. Sci. USA.

[22] Kračun S.K., Fangel J.U., Rydahl M.G., Pedersen H.L., Vidal-Melgosa S., Willats W.G.T., Lauc G., Wuhrer M. (2017). Carbohydrate Microarray Technology Applied to High-Throughput Mapping of Plant Cell Wall Glycans Using Comprehensive Microarray Polymer Profiling (CoMPP). High-Throughput Glycomics and Glycoproteomics: Methods and Protocols.

[23] Verhertbruggen Y., Marcus S.E., Haeger A., Ordaz-Ortiz J.J., Knox J.P. (2009). An Extended Set of Monoclonal Antibodies to Pectic Homogalacturonan. Carbohydr. Res..

[24] Willats W.G., Gilmartin P.M., Mikkelsen J.D., Knox J.P. (1999). Cell Wall Antibodies without Immunization: Generation and Use of de-Esterified Homogalacturonan Block-Specific Antibodies from a Naive Phage Display Library. Plant J..

[25] Liners F., Letesson J.-J., Didembourg C., Van Cutsem P. (1989). Monoclonal Antibodies against Pectin: Recognition of a Conformation Induced by Calcium. Plant Physiol..

[26] Ralet M.-C., Tranquet O., Poulain D., Moïse A., Guillon F. (2010). Monoclonal Antibodies to Rhamnogalacturonan I Backbone. Planta.

[27] Jones L., Seymour G.B., Knox J.P. (1997). Localization of Pectic Galactan in Tomato Cell Walls Using a Monoclonal Antibody Specific to (1[->]4)-[Beta]-D-Galactan. Plant Physiol..

[28] Moller I., Marcus S.E., Haeger A., Verhertbruggen Y., Verhoef R., Schols H., Ulvskov P., Mikkelsen J.D., Knox J.P., Willats W. (2008). High-Throughput Screening of Monoclonal Antibodies against Plant Cell Wall Glycans by Hierarchical Clustering of Their Carbohydrate Microarray Binding Profiles. Glycoconjucate J..

[29] Marcus S.E., Blake A.W., Benians T.A.S., Lee K.J.D., Poyser C., Donaldson L., Leroux O., Rogowski A., Petersen H.L., Boraston A. (2010). Restricted Access of Proteins to Mannan Polysaccharides in Intact Plant Cell Walls. Plant J..

[30] Pedersen H.L., Fangel J.U., McCleary B., Ruzanski C., Rydahl M.G., Ralet M.-C., Farkas V., von Schantz L., Marcus S.E., Andersen M.C.F. (2012). Versatile High Resolution Oligosaccharide Microarrays for Plant Glycobiology and Cell Wall Research. J. Biol. Chem..

[31] McCartney L., Marcus S.E., Knox J.P. (2005). Monoclonal Antibodies to Plant Cell Wall Xylans and Arabinoxylans. J. Histochem. Cytochem..

[32] Blake A.W., McCartney L., Flint J.E., Bolam D.N., Boraston A.B., Gilbert H.J., Knox J.P. (2006). Understanding the Biological Rationale for the Diversity of Cellulose-Directed Carbohydrate-Binding Modules in Prokaryotic Enzymes. J. Biol. Chem..

[33] Neumetzler L., Humphrey T., Lumba S., Snyder S., Yeats T.H., Usadel B., Vasilevski A., Patel J., Rose J.K.C., Persson S. (2012). The FRIABLE1 Gene Product Affects Cell Adhesion in Arabidopsis. PLoS ONE.

[34] Smallwood M., Beven A., Donovan N., Neill S.J., Peart J., Roberts K., Knox J.P. (1994). Localization of Cell Wall Proteins in Relation to the Developmental Anatomy of the Carrot Root Apex. Plant J..

[35] Pennell R.I., Janniche L., Kjellbom P., Scofield G.N., Peart J.M., Roberts K. (1991). Developmental Regulation of a Plasma Membrane Arabinogalactan Protein Epitope in Oilseed Rape Flowers. Plant Cell.

[36] Yates E.A., Valdor J.F., Haslam S.M., Morris H.R., Dell A., Mackie W., Knox J.P. (1996). Characterization of Carbohydrate Structural Features Recognized by Anti-Arabinogalactan-Protein Monoclonal Antibodies. Glycobiology.

