# Supplementary material for: Cell Wall Profiling of the Resurrection Plants Craterostigma plantagineum and Lindernia brevidens and Their Desiccation-Sensitive Relative, Lindernia subracemosa
Source: Plants (Basel). 2024 Aug 12;13(16):2235. doi: 10.3390/plants13162235 (PMC11359015; doi:10.3390/plants13162235)
Supplement: Supplementary file 1 [file plants-13-02235-s001.zip › Moore et al 2024 MDPI Plants Supplem Table1_2024-06-21.pdf]

Supplementary Material

Supplemental Table S1: Monoclonal antibodies and carbohydrate-binding modules used in this study

Monoclonal antibodies and carbohydrate-binding modules	Reference
HG partially/de-esterified (mAb JIM5)	[1]
HG partially esterified (mAb JIM7)	[1]
HG partially/de-esterified (mAb LM18)	[1]
HG partially/de-esterified (mAb LM19)	[1]
HG partially esterified (mAb LM20)	[1]
HG ±30 contiguous unmethylesterified GalA[a] units (mAb PAM1)	[2]
HG Ca^{2+} dimers (mAb 2F4)	[3]
RG-I, 6 unbranched disaccharide (mAb INRA-RU1)	[4]
RG-I, 2 unbranched disaccharide (mAb INRA-RU2)	[4]
β-1,4-D-galactan (mAb LM5)	[5]
α-1,5-L-arabinan (mAb LM6)	[2]
Linearised α-1,5-L-arabinan (mAb LM13)	[6]
β-1,4-D-(galacto)(gluco)mannan (mAb LM21)	[7]
β-1,4-D-(gluco)mannan (mAb LM22)	[7]
β-1,3-D-glucan (CBM BS-400-2)	[6]
Xyloglucan (XXXG motif) (mAb LM15)	[7]
Xyloglucan (XLLG oligosaccharide) (mAb LM24)	[8]
Xyloglucan (mAb LM25)	[8]
β-1,4-D-Xylan (mAb LM10)	[9]
β-1,4-D-Xylan d/arabinoxylan (mAb LM11)	[9]
Celulose (crystalline) (CBM3a)	[10]
Extensin (mAb LM1)	[11]
Extensin (mAb JIM11)	[12]
Extensin (mAb JIM20)	[12]
AGP (mAb JIM8)	[13]
AGP (mAb JIM13)	[14]
AGP (mAb LM14)	[6]
AGP,β-linked GlcA[b] (mAb LM2)	[14]

[a]GalA, galacturonic acid; [b]GlcA, glucuronic acid

References

1. Verhertbruggen, Y.; Marcus, S.E.; Haeger, A.; Ordaz-Ortiz, J.J.; Knox, J.P. An Extended Set of Monoclonal Antibodies to Pectic Homogalacturonan. *Carbohydrate Research* **2009**, *344*, 1858–1862, doi:10.1016/j.carres.2008.11.010.
2. Willats, W.G.; Gilmartin, P.M.; Mikkelsen, J.D.; Knox, J.P. Cell Wall Antibodies without Immunization: Generation and Use of de-Esterified Homogalacturonan Block-Specific Antibodies from a Naive Phage Display Library. *Plant Journal* **1999**, *18*, 57–65, doi:10.1046/j.1365-313x.1999.00427.x.
3. Liners, F.; Letesson, J.-J.; Didembourg, C.; Van Cutsem, P. Monoclonal Antibodies against Pectin: Recognition of a Conformation Induced by Calcium. *Plant Physiology* **1989**, *91*, 1419–1424, doi:10.1104/pp.91.4.1419.
4. Ralet, M.-C.; Tranquet, O.; Poulain, D.; Moïse, A.; Guillon, F. Monoclonal Antibodies to Rhamnogalacturonan I Backbone. *Planta* **2010**, *231*, 1373–1383, doi:10.1007/s00425-010-1116-y.

5. Jones, L.; Seymour, G.B.; Knox, J.P. Localization of Pectic Galactan in Tomato Cell Walls Using a Monoclonal Antibody Specific to (1[->]4)-[Beta]-D-Galactan. *Plant Physiology* **1997**, *113*, 1405–1412, doi:10.1104/pp.113.4.1405.
6. Moller, I.; Marcus, S.E.; Haeger, A.; Verhertbruggen, Y.; Verhoef, R.; Schols, H.; Ulvskov, P.; Mikkelsen, J.D.; Knox, J.P.; Willats, W. High-Throughput Screening of Monoclonal Antibodies against Plant Cell Wall Glycans by Hierarchical Clustering of Their Carbohydrate Microarray Binding Profiles. *Glycoconjucate Journal* **2008**, *25*, 37–48, doi:10.1007/s10719-007-9059-7.
7. Marcus, S.E.; Blake, A.W.; Benians, T.A.S.; Lee, K.J.D.; Poyser, C.; Donaldson, L.; Leroux, O.; Rogowski, A.; Petersen, H.L.; Boraston, A.; et al. Restricted Access of Proteins to Mannan Polysaccharides in Intact Plant Cell Walls. *Plant Journal* **2010**, *64*, 191–203, doi:10.1111/j.1365-313X.2010.04319.x.
8. Pedersen, H.L.; Fangel, J.U.; McCleary, B.; Ruzanski, C.; Rydahl, M.G.; Ralet, M.-C.; Farkas, V.; Schantz, L. von; Marcus, S.E.; Andersen, M.C.F.; et al. Versatile High Resolution Oligosaccharide Microarrays for Plant Glycobiology and Cell Wall Research *. *Journal of Biological Chemistry* **2012**, *287*, 39429–39438, doi:10.1074/jbc.M112.396598.
9. McCartney, L.; Marcus, S.E.; Knox, J.P. Monoclonal Antibodies to Plant Cell Wall Xylans and Arabinoxylans. *Journal of Histochemistry and Cytochemistry* **2005**, *53*, 543–546, doi:10.1369/jhc.4B6578.2005.
10. Blake, A.W.; McCartney, L.; Flint, J.E.; Bolam, D.N.; Boraston, A.B.; Gilbert, H.J.; Knox, J.P. Understanding the Biological Rationale for the Diversity of Cellulose-Directed Carbohydrate-Binding Modules in Prokaryotic Enzymes *. *Journal of Biological Chemistry* **2006**, *281*, 29321–29329, doi:10.1074/jbc.M605903200.
11. Neumetzler, L.; Humphrey, T.; Lumba, S.; Snyder, S.; Yeats, T.H.; Usadel, B.; Vasilevski, A.; Patel, J.; Rose, J.K.C.; Persson, S.; et al. The FRIABLE1 Gene Product Affects Cell Adhesion in Arabidopsis. *PLOS ONE* **2012**, *7*, e42914, doi:10.1371/journal.pone.0042914.
12. Smallwood, M.; Beven, A.; Donovan, N.; Neill, S. j.; Peart, J.; Roberts, K.; Knox, J. p. Localization of Cell Wall Proteins in Relation to the Developmental Anatomy of the Carrot Root Apex. *The Plant Journal* **1994**, *5*, 237–246, doi:10.1046/j.1365-313X.1994.05020237.x.
13. Pennell, R.I.; Janniche, L.; Kjellbom, P.; Scofield, G.N.; Peart, J.M.; Roberts, K. Developmental Regulation of a Plasma Membrane Arabinogalactan Protein Epitope in Oilseed Rape Flowers. *Plant Cell* **1991**, *3*, 1317–1326, doi:10.1105/tpc.3.12.1317.
14. Yates, E.A.; Valdor, J.F.; Haslam, S.M.; Morris, H.R.; Dell, A.; Mackie, W.; Knox, J.P. Characterization of Carbohydrate Structural Features Recognized by Anti-Arabinogalactan-Protein Monoclonal Antibodies. *Glycobiology* **1996**, *6*, 131–139, doi:10.1093/glycob/6.2.131.
